# Clinical outcomes after CPX‐351 in patients with high‐risk acute myeloid leukemia: A comparison with a matched cohort from the Spanish PETHEMA registry

**DOI:** 10.1002/cam4.6120

**Published:** 2023-05-22

**Authors:** Teresa Bernal, Ainhoa Fernández Moreno, Almudena de LaIglesia, Celina Benavente, Ana García‐Noblejas, Daniel García Belmonte, Rosalía Riaza, Olga Salamero, Maria Angeles Foncillas, Alicia Roldán, Víctor Noriega Concepción, Laura Llorente González, Juan Miguel Bergua Burgués, Soraya Lorente de Uña, Gabriela Rodríguez‐Macías, Adolfo de la Fuente Burguera, Maria José García Pérez, Jose Luis López‐Lorenzo, Pilar Martínez, Concepción Aláez, Marta Callejas, Carmen Martínez‐Chamorro, José Rifón Roca, Lourdes Amador Barciela, Armando V. Mena Durán, Karoll Gómez Correcha, Esperanza Lavilla Rubira, María Luz Amigo, Ferran Vall‐llovera, Ana Garrido, María García‐Fortes, Dunia de Miguel Llorente, Anastasia Aules Leonardo, Carlos Cervero, Rosa Coll Jordá, Manuel M. Pérez‐Encinas, Marta Polo Zarzuela, Angela Figuera, Guillermo Rad, David Martínez‐Cuadrón, Pau Montesinos

**Affiliations:** ^1^ Hospital Universitario Central Asturias Oviedo Spain; ^2^ Instituto de Oncología del Principado de Asturias (IUOPA), Instituto de Investigación del Principado de Asturias (ISPA) Spain; ^3^ Hospital Puerta de Hierro Madrid Spain; ^4^ Hospital Clínico San Carlos Madrid Spain; ^5^ Hospital La Princesa Madrid Spain; ^6^ Hospital Universitario Sanitas La Zarzuela Spain; ^7^ Hospital Universitario Severo Ochoa Madrid Spain; ^8^ Hospital Vall d'Hebrón Barcelona Spain; ^9^ Hospital Infanta Leonor Madrid Spain; ^10^ Departamento de Medicina Hospital Infanta Sofía San Sebastián de los Reyes, Universidad Europea Madrid Spain; ^11^ Complejo Hospitalario Universitario A Coruña A Coruña Spain; ^12^ Hospital Universitario HM Sanchinarro Madrid Spain; ^13^ Hospital San Pedro Alcántara Cáceres Spain; ^14^ Hospital Vithas Xanit Internacional Málaga Spain; ^15^ Hospital Gregorio Marañón Madrid Spain; ^16^ M.D. Anderson Cancer Center Madrid Spain; ^17^ Complejo Hospitalario Torre Cárdenas Almería Spain; ^18^ Hospital Universitario Fundación Jiménez Díaz Madrid Spain; ^19^ Hospital Universitario Doce de Octubre Madrid Spain; ^20^ Hospital Universitario La Moncloa Madrid Spain; ^21^ Hospital Universitario Príncipe Asturias Madrid Spain; ^22^ Hospital Universitario Quirón Pozuelo Madrid Spain; ^23^ Clínica Universitaria de Navarra Pamplona Spain; ^24^ Complejo Hospitalario Pontevedra Pontevedra Spain; ^25^ Consorcio Hospital General Universitario de Valencia Valencia Spain; ^26^ Hospital Juan Ramón Jiménez Huelva Spain; ^27^ Hospital Lucus Augusti Lugo Spain; ^28^ Hospital General Universitario Morales Messeguer Murcia Spain; ^29^ Hospital Mutua de Tarrasa Barcelona Spain; ^30^ Hospital de la Santa Creu i Sant Pau Barcelona Spain; ^31^ Hospital Universitario Virgen de la Victoria Málaga Spain; ^32^ Hospital Universitario de Guadalajara Guadalajara Spain; ^33^ Hospital Miguel Servet Zaragoza Spain; ^34^ Hospital Virgen de la Luz Cuenca Spain; ^35^ ICO Girona, Hospital Universitario Dr. Josep Trueta Girona Spain; ^36^ Hospital Universitario Santiago de Compostela Santiago Spain; ^37^ Hospital Universitari i Politècnic La Fe Valencia Spain

**Keywords:** acute myeloid leukemia, clinical observations, intensive chemotherapy, real‐world

## Abstract

**Background:**

CPX‐351 is approved for the treatment of therapy related acute myeloid leukemia (t‐AML) and AML with myelodysplastic related changes (MRC‐AML). The benefits of this treatment over standard chemotherapy has not been addressed in well matched cohorts of real‐life patients.

**Methods:**

Retrospective analysis of AML patients treated with CPX‐351 as per routine practice. A propensity score matching (PSM) was used to compare their main outcomes with those observed in a matched cohort among 765 historical patients receiving intensive chemotherapy (IC), all of them reported to the PETHEMA epidemiologic registry.

**Results:**

Median age of 79 patients treated with CPX‐351 was 67 years old (interquartile range 62–71), 53 were MRC‐AML. The complete remission (CR) rate or CR without recovery (CRi) after 1 or 2 cycles of CPX‐351 was 52%, 60‐days mortality 18%, measurable residual disease <0.1% in 54% (12 out of 22) of them. Stem cell transplant (SCT) was performed in 27 patients (34%), median OS was 10.3 months, and 3‐year relapse incidence was 50%. Using PSM, we obtained two comparable cohorts treated with CPX‐351 (*n* = 52) or IC (*n* = 99), without significant differences in CR/CRi (60% vs. 54%) and median OS (10.3 months vs. 9.1 months), although more patients were bridged to SCT in the CPX‐351 group (35% vs. 12%). The results were confirmed when only 3 + 7 patients were included in the historical cohort. In multivariable analyses, SCT was associated with better OS (HR 0.33 95% CI: 0.18–0.59), *p* < 0.001.

**Conclusion:**

Larger post‐authorization studies may provide evidence of the clinical benefits of CPX‐351 for AML in the real‐life setting.

## Graphical abstract

We report the results of CPX‐351 treatment in a group of 79 non‐selected patients reported to the PETHEMA epidemiologic registry and compared the main outcomes with a matched cohort among 765 historical patients receiving intensive chemotherapy. After propensity score matching, no differences in complete remission (60% vs. 54%) and median overall survival (10.3 months vs. 9.1 months) were observed between cohorts, although more patients were bridged to allogeneic transplant in the CPX‐351 group (35% vs. 12%). The results were confirmed when only 3 + 7 treated patients were included in the historical cohort.

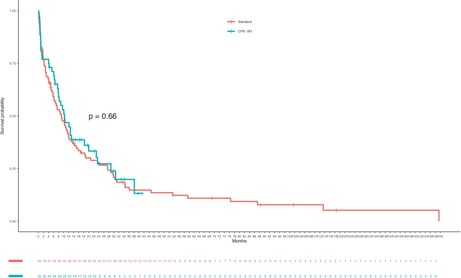

## 1 INTRODUCTION

CPX‐351, a liposomal formulation of cytarabine and daunorubicin, is approved for the treatment of therapy related acute myeloid leukemia (t‐AML) and AML with myelodysplastic related changes (MRC‐AML) according to WHO 2016 classification. This authorization is based on the results of a randomized phase 3 trial showing improved progression‐free and overall survival (OS) compared to standard intensive chemotherapy (IC).^1^

Several groups have reported results of CPX‐351 in non‐selected patients.^2^, ^3^, ^4^, ^5^ In only one of them a comparison with historical cohorts was made. In this study, a better OS was observed with CPX‐351 compared with a historical cohort of patients treated with IC. However, the groups were clinically and biologically different, and hence, the efficacy of the drug was not consistent across age ranges and genetic categories.^4^ Given the lack of evidence of the benefit of CPX‐351 over standard chemotherapy in real‐life, we first analyzed the clinical characteristics and main outcomes of a series of consecutive non‐selected patients treated with CPX‐351. Then, we investigated the Spanish PETHEMA registry to identify a cohort of patients with similar baseline characteristics who were treated with traditional chemotherapy schemes. Finally, the results on response rate, overall survival and relapse incidence were compared between cohorts.

## 2 METHODS

### 2.1 Patients

Patients who reported to the PETHEMA AML registry (NCT02607059) were included in this study if the following inclusion criteria were met: age ≥18‐year‐old; diagnosis of t‐AML or MRC‐AML and front‐line treatment with CPX‐351. The historical cohort comprised adult patients diagnosed with t‐AML or MRC‐AML who were registered between February 1984 to June 2020 and received front‐line intensive induction chemotherapy. First line AML patients treated with hypomethylating agents were excluded from the study.

Baseline clinical, biological and treatment variables were available for all patients. Response was assessed according to the International Working Group 2003 criteria.^6^

Minimal residual disease was assessed in CR/CRi patients by multi‐parameter flow cytometry (MFC). According to European Leukemia Net (ELN) measurable residual disease (MRD) working party guidelines, a 0.1% threshold was used to categorize MRD positive bone marrow samples.^7^, ^8^

### 2.2 Endpoints

The primary endpoint was overall survival. Secondary end points were percentage of CR/CRi, induction death, MRD negativity, rate of allogeneic stem cell transplant (SCT), cumulative incidence of relapse (CIR) and non‐leukemic death.

### 2.3 Statistical analysis

Baseline demographic, clinical and treatment related variables were summarized as median (interquartile range [IQR]) or frequency (proportion), as appropriate.

Overall survival was calculated from the first day of induction chemotherapy until the date of final follow‐up. Follow‐up was calculated with the observation time. Cumulative incidence of relapse was calculated from the moment of first remission until date of relapse accounting for the competing risk of death.^9^ SCT was analyzed as a time‐dependent variable.^10^

The probability of survival and differences between groups were estimated using the Kaplan–Meier method and the log‐rank test. A multivariable Cox model was performed to evaluate factors related to survival by including those variables with *p* value below 0.1 in univariate comparisons. In this model, hazard ratios (HRs) and 95% confidence intervals (95% CIs) were calculated.

Logistic regression was used to calculate propensity scores from baseline characteristics of age, gender, ECOG performance status, hematopoietic cell transplantation‐specific comorbidity index (HCTCI)^11^ and European Leukemia Net 2017 (ELN 2017) genetic risk.^7^ Propensity score matching with the nearest neighborhood method was used to match patients treated with CPX‐351 to those treated with IC.^12^ A 1:2 matching was used for the overall comparison. The absolute standardized mean differences (SMD) of the propensity scores of selected covariates were calculated before and after the match. A value <0.1 is indicative of adequate balance of covariate distribution between the two treatment groups.

Inverse probability of treatment weighting (IPTW) analysis based on the propensity scores was also performed on the pre‐matched cohort to assess OS between cohorts with the Cox proportional hazard model.

All the statistical analyses were performed using the R statistical package (version 4.2.0).

## 3 RESULTS

### 3.1 CPX‐351 treated patients

Overall, 85 patients with t‐AML or AML‐MRC received induction with CPX‐351. In 6 of them, first line HMA were used for AML treatment and were excluded from the analysis. Median age of the remaining 79 patients was 67 (IQR 62–71) years (range 20–78). Sixty seven percent of the patients (53/79) were classified as AML‐MRC. Among the 53 AML‐MRC patients, the diagnosis was based on morphological detection of multilineage dysplasia in 12, whereas 16 patients had previous history of MDS‐related. MDS cytogenetic abnormalities were present in 25/53 patients. ELN 2017 genetic risk category was favorable, intermediate, adverse and indeterminate in 11% (9/79), 38% (30/79), 46% (36/79) and 5% (4/79) patients, respectively. Twenty eight percent patients (22/79) had received prior cytotoxic treatment. Among them, 36% (8/22) harbored ELN2017 adverse genetics, 95% (21/22) had HCTCI score ≥3, and 59% (13/22) were not transplanted. Table S1 shows the baseline characteristics of the population.

### 3.2 Response assessment

Percentage of CR/CRi after one induction cycle was 43% (34/79). Eleven patients received a second CPX‐351 cycle after the failure of the first one. Seven out of the 11 (64%) non‐responding patients who received a second cycle with CPX‐351 achieved first CR/CRi, resulting in a CR/CRi rate after 1 or 2 cycles of 52%. Additionally, 15 non responders received other second induction therapy including FLUGA (*n* = 1), FLAG‐ida (*n* = 3), HMA alone (*n* = 4) or in combination with venetoclax (*n* = 4), HDAC (*n* = 2), mini‐MEC (mitoxantrone, etoposide and cyclophosphamide, *n* = 1). Rate of CR/CRi among these patients was 20% (3/15).

MRD was evaluable after first cycle in 65% (22/34) responding patients and was below 0.1% in 54% (12/22) of them. Detailed information regarding treatment response and toxicity is shown in Table 1.

**TABLE 1:** Treatment response and toxicity after first induction cycle.

Characteristics	*n*	%
Percentage of CR/CRi		
After 1st CPX‐351	34	43
+ After 2nd CPX‐351	7	52
+ After other 2nd line of treatment	3	56
Median time to ANC recovery (days)		
≥0.5 × 10^9^/L	31 (27–42)	
≥1 × 10^9^/L	35 (31–44)	
Median time to platelet recovery (days)		
≥50 × 10^9^/L	31 (26–41)	
≥100 × 10^9^/L	39 (31–43)	
Death in induction	14	18
After 30 days	10	13
After 60 days	4	5
Cause of death		
Infection	9	64
Hemorrhage	4	29
Veno‐oclusive disease	1	7

Abbreviations: ANC, absolute neutrophil count; CR, complete remission; CRi, complete remission with incomplete peripheral blood recovery.

Median age of CR/CRi patients was 67 years (65–72) compared to 66 (66–71) years in non‐responding patients, *p* = 0.30. Univariable analyses showed that no variable was associated with response after 1 cycle (Table S2).

### 3.3 Allogeneic stem cell transplant

SCT was performed in 34% (27/79) patients after a median of 4.5 (range 2.9–5.8) months from the beginning of induction. The median number of cycles, including induction and consolidation, before transplant were 2 (range 1–2). Disease status at SCT was CR/CRi (*n* = 22), MLFS (*n* = 2) and active disease (*n* = 3). Sixteen patients (59%) received alternative donor source (15 haploidentical and 1 umbilical cord blood), whereas the remaining patients received matched unrelated or sibling donors. Reduced intensity conditioning was used in 63% (17/27) patients.

**FIGURE 1:**
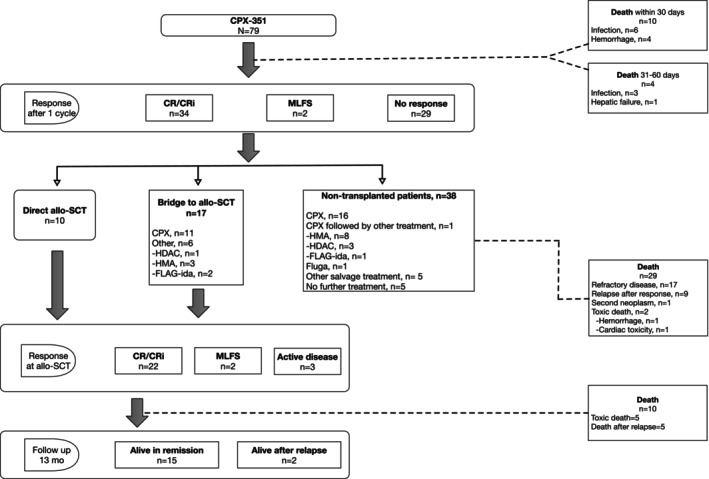
Flow chart of patients treated with CPX‐351.

The complete flow‐chart of patients and their treatments is shown in Figure 1.

### 3.4 Survival

With a median follow up of 8.9 months (IQR: 3.2–21.1) in the whole population and 24.6 months (IQR: 16.3–33) in alive patients, 54 events occurred, resulting in a median OS of 10.3 months (95% CI: 7.6–19.7). In univariable analyses, significant differences were observed between transplanted (OS = NR [95% CI: 12.7–NR]) and non‐transplanted patients (OS 5.4 months [95% CI 2.5–12.7]), HR 0.23 (95% CI 0.1–0.46), (Figure S1). Baseline characteristics associated with better survival were age younger than 65 years (*p* = 0.002), female gender (*p* = 0.007), ECOG performance status 0–1 (*p* = 0.008), low or intermediate HCTCI comorbidity index (*p* = 0.02), non‐MRC‐AML WHO category (*p* = 0.02), and ELN 2017 favorable genetic risk (*p* = 0.02) (Table S3). In multivariable analysis considering SCT as a time dependent covariate, factors related to OS were SCT [HR 0.16 (95% CI: 0.07–0.36), *p* < 0.001]; ECOG PS ≥2 [HR 4.15 (95% CI 1.59–10.8), *p* = 0.002], age older than 65 years [HR 3.29 (95% CI 0.07–0.36), *p* = 0.002] and male sex [HR 046 (95% CI 0.22–0.95), *p* = 0.03 (Table 2).

**TABLE 2:** Multivariable analysis for overall survival in CPX‐351 treated patients.

	HR (95% CI)	*p*
Age >65 years old	3.29 (1.52–7.14)	0.002
Male	0.46 (0.22–0.95)	0.03
ECOG PS ≥2	4.15 (1.59–10.8)	0.003
HCTCI‐CI ≥3	0.42 (0.13–1.35)	0.14
Recurrent genetic abnormalities	0.36 (0.05–2.66)	0.7
ELN 2017 favorable category versus others	0.37 (0.05–2.35)	0.29
Therapy‐related	1.2 (0.27–14.48)	0.49
SCT	0.16 (0.07–0.36)	<0.001

Abbreviations: ECOG, Eastern Cooperative Oncology Group; ELN17, European Leukemia Net 2017 genetic risk classification; HCTCI, Hematopoietic Cell Transplant Comorbidity Index; MRC: Myelodysplastic Related Changes; NOS, Not Otherwise Specified; WHO, World Health Organization.

### 3.5 Cumulative incidence of relapse

Responding patients were followed during a median of 16.5 (IQR 7.3–28.1) months after achieving first CR/CRi. In these patients, CIR at 3 years was 50% (95% CI 35–70). In multivariable analysis, SCT was significantly associated with lower CIR: HR 0.08 (95% CI 0.03–0.27), *p* < 0.001. Conversely, ELN17 adverse genetic risk was associated with higher CIR, although this association did not reach statistical significance: HR 2.85 (95% CI 0.95–8.57), *p* = 0.06. The results of multivariable analysis of CIR are provided on detail in Table S4.

### 3.6 Propensity score matching

The historical control arm was based on 765 unselected t‐AML and MRC‐AML patients registered in the PETHEMA database receiving front‐line IC. These patients were diagnosed with AML from 1984 to 2019. Only 2 patients from the historical cohort were diagnosed in 2020. In this cohort, the rate of allogeneic transplant ranged from 5% before 2010 to 13% between 2010 and 2018. In 2019, 47 AML patients were reported to the registry and 9 of them (19%) were transplanted.

Before matching, baseline characteristics differed significantly between the CPX‐351 and t‐AML/MRC‐AML historical cohorts (Table S5). After the propensity score matching, SMDs of all covariates were <0.1, showing adequate balance between the control and the CPX‐351 cohorts, as it is shown in Table S6 and Figure S2.

The median age of 99 controls and 52 CPX‐351 treated patients was 67 (64–71) and 68 (65–71) years, respectively. The proportion of patients with poor performance status, high HCTCI and ELN 2017 adverse genetic risk category were similar between control and CPX‐351 treated patients. Within each arm, there were 11% (11/99) and 15% (8/52) patients with ECOG ≥2, 19% (19/99) and 25% (13/52) patients with HCTCI ≥2 and 52% (52/99) and 50% (26/52) patients with ELN17 adverse genetics, respectively. Table S5 shows the univariable comparisons of baseline characteristics between control and CPX‐351 treated patients. Chemotherapy regimens used in the control arm are shown in Table S7.

### 3.7 Responses in the CPX‐351 versus the matched historical control arm

CR/CRi rate after one or two induction cycles was similar between historical IC control and CPX‐351 arms, with 54% (53/99) and 60% (31/52), respectively, *p* = 0.3, Table S8.

Information on MRD was available in 30% (16/53) responding patients in control arm and 61% (19/31) patients in CPX‐351 arm, being negative in 50% (8/16) of the control and 58% (11/19) of the CPX‐351 group patients, respectively.

SCT was performed in 12% (12/99) control patients and 35% (18/52) CPX‐351 patients. In the former group, autologous SCT was performed in four patients with ELN 2017 favorable (*n* = 2) and intermediate genetic risk (*n* = 2).

### 3.8 Overall Survival in the CPX‐351 versus the matched historical control arm

**FIGURE 2:**
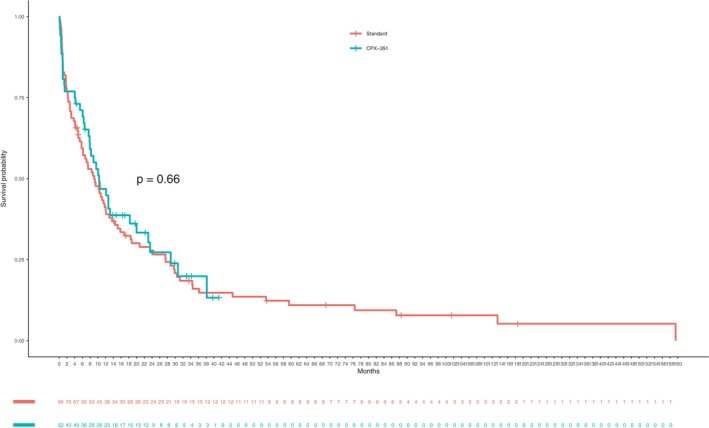
Overall survival according to induction chemotherapy.

With a median follow up of 8.9 months (IQR 2.5–20.4), no differences in OS were observed between control [9.1 months (95% CI 6.2–13)] and CPX‐351 arm [10.3 months (95% CI 7.9–23)], Log Rank *p* = 0.66 (Figure 2).

In multivariable analysis of OS performed in the matched cohorts including age, ECOG performance status, HCTCI comorbidity index, ELN17 risk category and SCT, only SCT was associated with better OS (HR 0.33 95% CI: 0.18–0.59), *p* < 0.001. The inclusion of induction arm in the multivariable model did not show a significant effect of CPX‐351 on survival (HR 1.01 95% CI 0.7–1.47), *p* = 0.94. The uni‐ and multivariable analysis of overall survival in the matched cohorts are shown in Table 3 and Figure S3.

**TABLE 3:** Univariable and multivariable analysis of overall survival in the matched cohorts.

	Univariate analysis		Multivariable analysis	
	Median OS	Log‐rank *p*	HR (95% CI)	*p*
Age, ≥70 years				
No	10.5 (8.2–16.8)	0.03	1 0.03 (0.7–1.50)	0.86
Yes	7.5 (4.1–12)			
ECOG PS ≥2				
No	10.3 (7.9–13.7)	0.04	1.27 (0.76–2.11)	0.36
Yes	6.2 (0.9–15.1)			
HCTCI‐CI				
Low/intermediate	10.2 (7.9–12.7)	0.6	1.21 (0.76–1.93)	0.41
High	6.2 (3.1–18.3)			
European Leukemia Net 2017 genetic risk category				
Favorable	23.5 (13–NR)	0.05	0.52 (0.27–1.01)	0.05
Intermediate	8.4 (4.1–20)			
Adverse	9.4 (7–12)			
NA	2.9 (0.6–NR)			
SCT	6.5 (4.9–9.6) 28.7 (23–NA)	<0.001	0.33 (0.18–0.59)	<0.001

Abbreviations: ECOG, Eastern Cooperative Oncology Group; ELN17, European Leukemia Net 2017 genetic risk classification; Yo, years old.

Since there was a substantial heterogeneity in the chemotherapeutic schemes used in the historical cohort, the PSM was repeated including only those patients of the historical cohort receiving standard 3 + 7 scheme. This scheme, consisting of idarubicin 12 mg/m^2^ over 3 days and cytarabine 200 mg/m^2^ over 7 days, was administered to 377 patients. After PSM, the matched cohort comprised 110 patients treated with standard 3 + 7 chemotherapy and 63 patients treated with CPX‐351. The median OS in the control arm was 10.3 months (95% CI 7.9–23) and 8 months (95% CI 5.8–1.7) in the CPX‐351 arm, *p* = 0.28. Table S9 shows the SMDs of covariates after the new PSM. Figure S4 shows the comparison of OS between the 2 cohorts.

Finally, we performed an IPTW analysis in the pre‐matched cohorts of CPX‐351 and standard 3 + 7 treated patients to assess OS between cohorts. In this analysis, the median OS in the control arm was 7.8 months (95% CI 6.4–9.6) and 8.9 months (95% CI 6–19.7) in the CPX‐351 arm, *p* = 0.47. Figure S5 shows the comparison of OS between the 2 cohorts.

### 3.9 Cumulative incidence of relapse in the CPX‐351 versus the matched historical control arm

**FIGURE 3:**
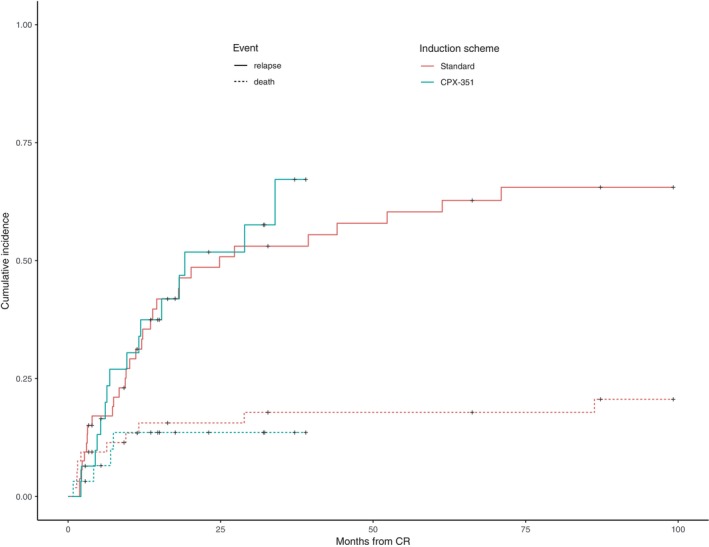
Cumulative incidence of relapse and death in the matched cohort.

In the matched cohorts, median follow up of CR/CRi patients was 14.8 months (IQR: 6.6–31.2). CIR at 3 years was 58% (95% CI 54–71) without differences between matched control and CPX‐351 treated patients (Figure 3). In multivariable analysis, SCT was significantly associated with lower relapse incidence: HR = 0.25 (95% CI: 0.1–0.6), *p* < 0.003, but ELN17 adverse genetic category and treatment arm did not: HR = 1.19 (95% CI: 0.6–2.12), *p* = 0.53 and HR = 1.48 (95% CI: 0.83–2.65), *p* = 0.17, respectively.

## 4 DISCUSSION

In this study, we have analyzed the real‐life outcomes of a group of AML patients treated with CPX‐351 as per routine practice under the FDA/EMA approved indications and compared their main outcomes with those observed in a matched cohort of historical patients treated with conventional intensive chemotherapeutic regimens. Unlike the pivotal phase 3 trial leading to CPX‐351 regulatory approval, we did not find significant improvements in the CPX‐351 as compared to the IC cohort. These findings were consistent after excluding from the historical cohort those patients not treated with the standard 3 + 7 scheme.

As compared to other similar studies from German, French, or Italian groups, we report here worse real‐life outcomes using CPX‐351, with lower CR/CRi rates (except for German study) and OS estimates.^3^, ^4^, ^5^ These differences could be explained, at least in part, by the characteristics of our CPX‐351 cohort, where the vast majority of patients had 60 years or more (IQR 62–71 years old). Another explanation for our shorter OS could be the higher rate of SCT performed in other real‐life studies with CPX‐351, especially in the German series.^5^ We can speculate that, in our context, CPX‐351 was used in a population more similar to that enrolled in the pivotal phase 3 trial (60–75 years old) than that for FDA/EMA label indication. In fact, the CR/CRi rate (52%) and SCT rate (34%) among 79 unselected patients treated with CPX‐351 in the PETHEMA group were aligned with those reported by the pivotal phase 3 trial. Interestingly, we found similar CR/CRI rates after CPX‐351 when comparing our series and phase 3 trial among specific subsets, such as t‐AML (47% vs. 50%), ELN17 adverse genetic risk (43% vs. 39%), and previous HMA exposure (37% vs. 38%). Moreover, the 10.3 months OS in our patients overlaps with the 9.5 months survivals of the CPX‐351 phase 3 arm. We need to highlight that all patients treated with CPX‐351 in our study period were included, even if they were not evaluable for response due to early complications (to receive 1 dose of CPX‐351 was enough to be selected). As a possible consequence, we found 60‐days mortality rate of 18%, higher than the 13% published by Lancet et al.^1^ This finding is not surprising, since cancer patients included in clinical trials do not accurately represent the general population.^13^ On the other hand, the longer duration of neutropenia and thrombocytopenia after CPX‐351 administration compared to “3 + 7” IC scheme favors infection and hemorrhage, were the main causes of death in our cohort.

As the selection of patients must be considered as a principal driver for outcomes in clinical studies, we aimed to compare CPX‐351 outcomes with a matched historical cohort using propensity score method. The analysis of covariate balance confirmed the adequacy of the matching and the reduced risk of selection bias. Indeed, no difference were observed in baseline characteristics between groups, including a 50% rate of ELN17 adverse genetics in both cohorts. Although we could not demonstrate significant differences in CR/CRi, MRD, OS, and CIR between both matched arms, we underline that our analysis is far from the best methodology to explore differences between treatment options. However, although matching by well‐established prognostic features, it was not possible to capture subtle differences between CPX‐351 cohort and conventional chemotherapy comparator group, potentially leading to unexpected different outcomes. As of today, we must rely on well‐designed randomized trials to set new indications and demonstrate therapeutic improvements. Nonetheless, these improvements should be challenged in post‐authorization studies, like this one.

It must be noted that the 34% CR/CRi rate observed in the standard arm in the phase 3 clinical trial^1^ was quite below the 50% rate observed in most studies focusing on secondary AML.^14^, ^15^, ^16^, ^17^ Therefore, we can argue that suboptimal results in the control arm of the pivotal phase 3 trial favored comparison with CPX‐351. On the other hand, the cytarabine dose administered in the clinical trial was 100 mg/m^2^, whereas 90% patients treated in our IC control arm of the historical cohort received 200 mg/m^2^ or even high dose cytarabine. Whether lower cytarabine dose‐intensity in the phase 3 control‐arm may have contributed to suboptimal outcomes remains unclear.

We found high relapse incidence after achieving remission both in the matched CPX‐351 and historical cohorts, and relapse rate was not modified by any variable, with the exception of SCT. It is intriguing that, even when more patients treated with CPX‐351 were transplanted, no differences in relapse were observed between both cohorts. However, the low number of patients transplanted globally precludes to perform further analysis.

One important constraint of our study is the small number of patients treated with CPX‐351 compared to the phase 3 trial. This might have limited the statistical power to confirm or refute the efficacy of CPX‐351. In addition, our study lacks information regarding myelodysplastic‐related gene mutations or prior hypomethylating agent exposure in the historical cohort. However, it must be noted that in the phase 3 trial, patients were stratified by cytogenetics, but not by gene mutations.

In conclusion, this retrospective analysis performed in t‐AML and AML‐MRC patients treated with front‐line CPX‐351 shows that the phase 3 results are reproducible in routine practice. However, the comparison with a synthetic retrospective cohort receiving conventional chemotherapy has failed to prove any superiority. It is desirable that larger post‐authorization studies are performed in the real‐life setting to provide evidence of the clinical benefits of CPX‐351 for t‐AML and AML‐MRC.

## AUTHOR CONTRIBUTIONS

**Teresa Bernal:** Conceptualization (lead); data curation (lead); formal analysis (lead); investigation (lead); methodology (lead); project administration (lead); writing – original draft (lead); writing – review and editing (lead). **Ahinoa Fernandez Moreno:** Investigation (supporting); validation (supporting); visualization (equal). **Almudena de Laiglesia:** Investigation (supporting); validation (supporting); visualization (supporting). **Celina Benavente:** Investigation (supporting); validation (supporting). **Ana García‐Noblejas:** Investigation (supporting); validation (supporting). **Daniel García‐Belmonte:** Investigation (supporting); validation (supporting). **Rosalía Riaza:** Investigation (supporting); validation (supporting). **Olga Salamero:** Investigation (supporting); validation (supporting). **Maria Angeles Foncillas:** Investigation (supporting); validation (supporting). **Alicia Roldán:** Investigation (supporting); validation (supporting). **Victor Noriega:** Investigation (supporting); supervision (equal); validation (supporting). **Laura Llorente Gonzalez:** Investigation (supporting); validation (supporting). **Juan Miguel Bergua Burgues:** Investigation (supporting); validation (supporting). **Soraya Lorente de Uña:** Investigation (supporting); validation (supporting). **Gabriela Rodriguez Macías:** Investigation (supporting); validation (supporting). **Adolofo de la Fuente Burguera:** Investigation (supporting); validation (supporting). **Maria Jose Garcia Perez:** Investigation (supporting); validation (supporting). **Jose Luis Lopez Lorenzo:** Investigation (supporting); validation (supporting). **Pilar Martinez‐Sanchez:** Investigation (supporting); validation (supporting). **Concepción Alaez:** Investigation (supporting); validation (supporting). **MARTA CALLEJAS:** Investigation (supporting); validation (supporting). **Carmen Martinez Chamorrro:** Investigation (supporting); validation (supporting). **Jose Rifon Roca:** Investigation (supporting); validation (supporting). **Lourdes Amador Barciela:** Investigation (supporting); validation (supporting). **Armando V Mena Durán:** Investigation (supporting); validation (supporting). **Karoll Gomez Correcha:** Investigation (supporting); validation (supporting). **Esperanza Lavilla Rubira:** Investigation (supporting); validation (supporting). **María Luz Amigo:** Investigation (supporting); validation (supporting). **Ferran Vall‐llovera:** Investigation (supporting); validation (supporting). **Ana Garrido:** Investigation (supporting); validation (supporting). **María Garcia‐Fortes:** Investigation (supporting); validation (supporting). **Dunia de Miguel Llorente:** Investigation (supporting); validation (supporting). **Anastasia Aules Leonardo:** Investigation (supporting); validation (supporting). **Cerveró Carlos:** Investigation (supporting); validation (supporting). **Rosa Coll:** Investigation (supporting); validation (supporting). **Manuel Pérez‐Encinas:** Investigation (supporting); validation (supporting). **Marta Polo:** Investigation (supporting); validation (supporting). **Angela Figuera:** Investigation (supporting); validation (supporting). **Guillermo Rad:** Formal analysis (supporting); investigation (supporting). **David Martínez‐Cuadrón:** Investigation (supporting); software (equal); validation (supporting). **Pau Montesinos:** Conceptualization (lead); funding acquisition (lead); project administration (lead); writing – review and editing (lead).

## FUNDING INFORMATION

This study was funded in part by a research grant from the Jazz Pharmaceuticals.

## ETHICS AND INTEGRITY

This Study was approved by the Hospital Universitario Central de Asturias Research Ethics Board according to the Declaration of Helsinki. Informed consent was a requisite for patients alive at the time of the analyses.

## Supporting information


Data S1.

## Data Availability

The datasets generated during and/or analyzed during the current study are available from the corresponding author on reasonable request.
