# Supplementary material for: Clinical outcomes after CPX‐351 in patients with high‐risk acute myeloid leukemia: A comparison with a matched cohort from the Spanish PETHEMA registry
Source: Cancer Med. 2023 May 22;12(14):14892–901. doi: 10.1002/cam4.6120 (PMC10417130; doi:10.1002/cam4.6120)
Supplement: Supplementary file 1 — Data S1. [file CAM4-12-14892-s001.pdf]

Supplementary Table 1. Baseline characteristics of front-line treated patients with CPX-351 (n=79).

	N	%
**Age**, years (median, IQR)	67 (62-71)	
**Sex**		
Female	34	43
Male	45	57
**ECOG PS** (n=67)		
0-1	58	87
≥2	9	13
**HCTCI**		
Low	21	27
Intermediate	24	30
High	34	43
**Previous neoplasm**	57	72
MDS/MDS-MPN	26	33
MPN	6	7
Solid	25	32
**WHO 2016 category**		
MRC	53	67
Recurrent genetic abnormalities	10	13
Post-cytotoxic treatment	13	16
NOS	3	4
**Previous cytotoxic treatment**	22	27
**ELN 2017 genetic risk**		
Favorable	9	11
Intermediate	30	38
Adverse	36	46
NA*	4	5
**WBC at diagnosis** x10^9/L (median, IQR)	5 (2.2-13.4)	
**Pretreatment with HMA**	13	16

ELN17: European Leukemia Net 2017 genetic risk classification. ECOG: Eastern Cooperative Oncology Group Performance Status. WHO: World Health Organization. MRC: Myelodysplastic Related Changes. NOS: Not Otherwise Specified. HCTCI: Hematopoietic Cell Transplant Comorbidity Index.

*Two patients with antecedent of high risk MDS. Two patients with therapy related with FAB M7 morphology (1) and dry aspirate (1)

Supplementary Table 2. Univariable analysis of response after one cycle.

	N (%)	P
**ECOG PS** (n=58)		
• 0-1	9/57 (51)	0.1
• ≥2	3/9 (33)	
**Gender**		
• Male	15/44 (34)	0.2
• Female	19/34 (56)	
**WHO 2016 category**		
• MRC	20/53 (38)	
• Therapy-related	5/13 (38)	0.2
• Not otherwise specified	2/3 (67)	
• Recurrent genetic abnormalities	7/10 (70)	
**ELN17 genetic risk category**		
• Favorable	6/9 (66)	
• Intermediate	11/30 (37)	0.2
• Unfavorable	14/35 (40)	
• NA	3/4 (75)	
**Prior cytotoxic treatment**		
• No	33/56 (40)	0.6
• Yes	11/22 (50)	
**Pretreatment with HMA**		
• Yes	5/12 (42)	1
• No	29/66 (44)	

ELN17: European Leukemia Net 2017 genetic risk classification. ECOG PS: Eastern Cooperative Oncology Group Performance Status. WHO: World Health Organization. MRC: Myelodysplastic Related Changes. NOS: Not Otherwise Specified. HCTCI: Hematopoietic Comorbidity Charlson Index.

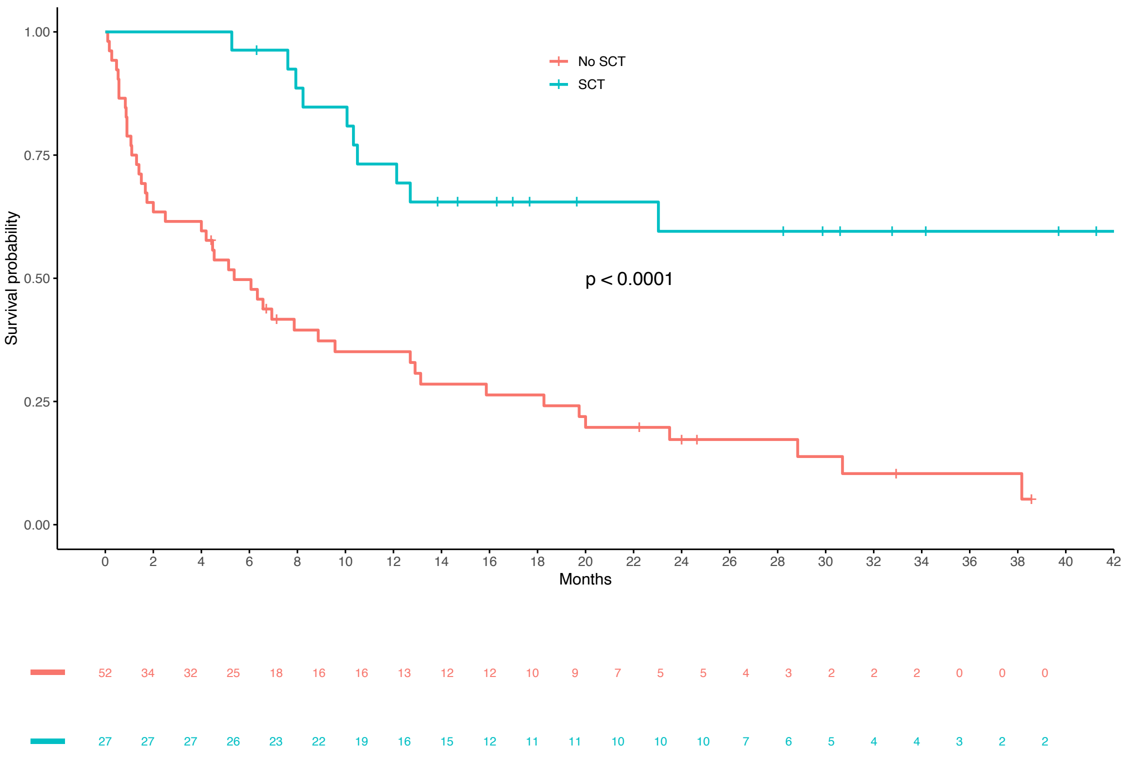

Supplementary Figure 1. Overall survival according to Stem Cell Transplant (SCT).

Supplementary Table 3. Univariable analysis of overall survival.

	OS (months)	P
**Age, ≥ 65 years**		
• No	28.8 (12.9-NR)	0.002
• Yes	7.9 (4.13-12.7)	
**Sex**		
• Female	19.7 (12.7-NR)	0.007
• Male	6.3 (2.5-10.3)	
**ECOG PS** (n=67)		
• 0-1	12.7 (8.9-23.5)	0.008
• ≥2	0.86 (0.46-NR)	
**HCTCI-CI**		
• Low/intermediate	8.2 (5.13-12.7)	0.02
• High	18.2 (7.8-NR)	
**WHO 2016 category**		
• MRC	7.9 (6-12.7)	0.04
• Therapy-related	13 (5.2-NR)	
• Not otherwise specified	20 (2.5-NR)	
• Recurrent genetic abnormalities	38.2 (23.5-NR)	
**Previous cytotoxic treatment**		
• No	8.2 (5.1-12.7)	0.007
• Yes	30.7 (13.1-NR)	
**ELN17 genetic risk category**		
• Favorable	NR (38.2-NR)	0.02
• Intermediate	7.9 (2.5-NR)	
• Adverse	8.9 (5.3-18.3)	
• NA	7.9 (0.26-NR)	
**WBC at diagnosis ≥50 x10^9/L**		
• No	12.7 (7.9-23.5)	0.2
• Yes	8.2 (5.1-NR)	
**Pretreatment with HMA**		
• No	12.7 (7.9-23)	0.4
• Yes	6.9 (1.3 NR)	

ELN17: European Leukemia Net 2017 genetic risk classification. ECOG PS: Eastern Cooperative Oncology Group Performance Status. WHO: World Health Organization. MRC: Myelodysplastic Related Changes. NOS: Not Otherwise Specified. HCTCI: Hematopoietic Cell Transplant Comorbidity Index.

Supplementary Table 4. Multivariable analysis for cumulative incidence of relapse

	**HR (95% CI)**	**P**
**Age >65 yo**	1.60 (0.33-7.58)	0.55
**HCTCI-CI ≥3**	0.39 (0.13-1.19)	0.10
**WHO 2016 MRC**	2.33 (0.67-8.0)	0.18
**ELN 17 adverse category**	2.85(0.95-8.57)	0.06
**SCT**	0.08 (0.03-0.27)	<0.0001

Yo: years old. HCTCI: Hematopoietic Cell Transplant Comorbidity Index. WHO 2016 MRC: World Health Organization Myelodysplastic related changes category. ELN17: European Leukemia Net 2017 genetic risk classification. NA: not available.

Supplementary Table 5. Univariable comparisons of baseline characteristics in the pre-matched (left) and matched (right) cohorts.

	Pre-matching N (%)			Post-matching N (%)		
	**Historical control (N=765)**	**CPX-351 (N=79)**	**P**	**Historical control (N=99)**	**CPX-351 (N=52)**	**P**
**Age**	66 (63-70)	67 (62-71)	0.9	67 (64-71)	68 (65-71)	0.8
**Sex male**	299/765 (39)	45/79 (57)	0.6	47/99 (47)	26/52 (50)	0.9
**ECOG-PS ≥1**	145/676 (21)	9/67 (13)	0.2	11 (11/99)	15 (8/52)	0.6
**HCTCI >2**	158/765 (20)	26/79 (34)	0.02	19 (19/99)	25 (13/52)	0.5
**ELN17**<br>• Favorable<br>• Intermediate<br>• Adverse<br>• NA	 36/765 (5)<br>107/765 (14)<br>529/765 (69)<br>93/765 (12)	 9/79 (11)<br>30/79 (38)<br>36/79 (46)<br>4/79 (5)	<.001	 12/99 (12)<br>31/99 (31)<br>52/99(52)<br>4/99 (40)	 5/52(9)<br>19/52(37)<br>26/52(50)<br>2/52(4)	0.9

ECOG-PS: Eastern Cooperative Oncology Group Performance Status. HCTCI: Hematopoietic Cell Transplant Comorbidity Index. ELN17: European Leukemia Net 2017 genetic risk classification. NA: not available

Supplementary Table 6. Mean Standard Differences (MSD) of covariates before and after matching.

Baseline characteristics	Before	After
**Age**	0.446	0.024
**Sex**	0.414	-0.019
**ECOG ≥2**	0.180	0.048
**HCTCI ≥3**	0.104	0.038
**ELN17 (%)**<br>• Favorable<br>• Intermediate<br>• Adverse<br>• NA	0.710	 -0.019<br>0.019<br>0<br>0

ECOG: Eastern Cooperative Oncology Group. ELN17: European Leukemia Net 2017 genetic risk classification.

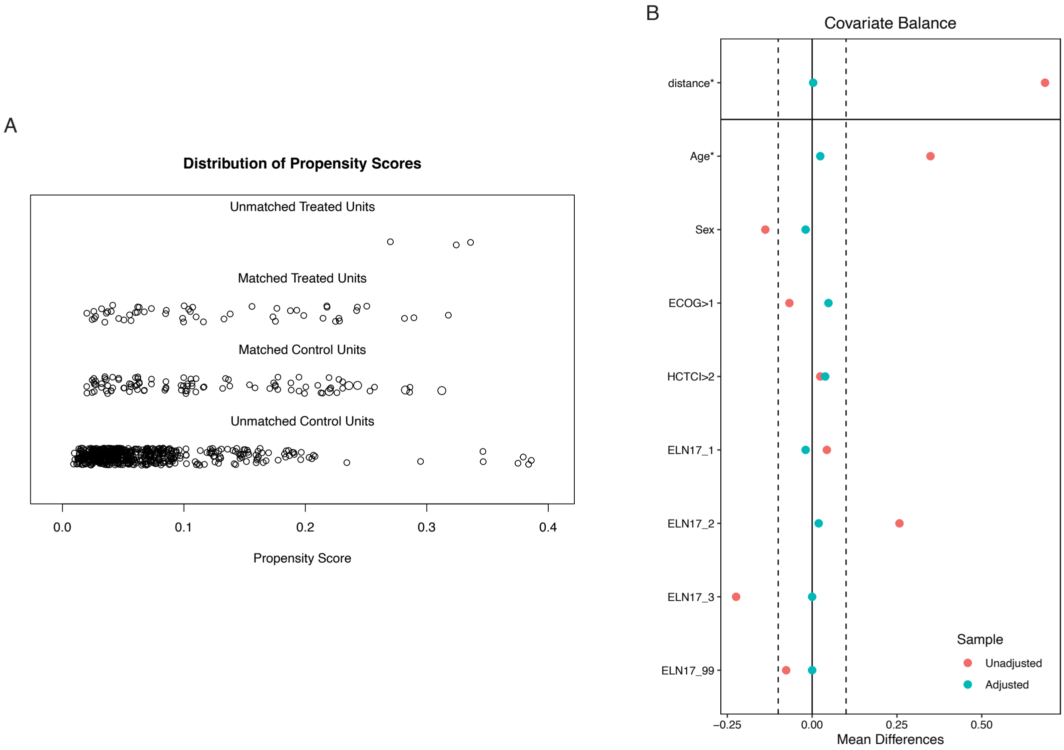

Supplementary Figure 2. A) Distribution of propensity scores in unmatched and matched cohorts. B) Covariate balance measured by standardized mean difference.

Supplementary Table 7. Chemotherapy regimens used in the historical matched cohort.

Chemotherapy	Nº of patients	Description
Idarubicin + cytarabine	43	Idarubicin 12 mg/m^2 days 1-3<br>Cytarabine 200 mg/m^2 days 1-7
Idarubicin + cytarabine	16	Idarubicin 12 mg/m^2 days 1-2<br>Cytarabine 200 mg/m^2 days 1-5
Daunorubicin + cytarabine	2	Daunorubicin 60 mg/m^{2}x days 1-3<br>Cytarabine 200 mg/m^2 days 1-7
Idarubicin + cytarabine	13	Idarubicin 8 mg/m^2 days 1-3<br>Cytarabine 100 mg/m^2 days 1-7
Mitoxantrone + cytarabine	2	Mitoxantrone 12 mg/m^2 days 1-3<br>Cytarabine 100 mg/m^2 days 1-7
ICE	2	Idarubicin 12 mg/m^2 days 1,3,5 Cytarabine 500 mg/m^2/12h days 1,3,5,7 Etoposide 100 mg/m^2 days 1-3
FLAG-IDA	13	Fludarabine 30 mg/m2/d days 1-3<br>Idarubicin 10 mg/m2/d days 1-3<br>Cytarabine 2 g/m2/d days 1-4<br>G-CSF5 mcg/d days 1-5
FLAG	1	Fludarabine 30 mg/m2/d days 1-3<br>Cytarabine 2 g/m2/d days 1-4<br>G-CSF5 mcg/d days 1-5
FLA	1	Fludarabine 30 mg/m2/d days 1-3<br>Cytarabine 2 g/m2/d days 1-4
Miscellaneous<br>1. DAT<br>2. Cytarabine-Thioguanine - Mitoxantrone<br>3. NOVE<br>4. Cytarabine<br>5. Unknown	 1<br>1<br><br>1<br>2<br>1	1. DAT: Daunorubicin 60 mg/m^{2}x days 1-3 +Cytarabine 200 mg/m^2 days 1-5 +Thioguanine 40 mg/m$^{2\ days}$ 2-5<br>2. Cytarabine 600 mg/m^2 day 1 +Thioguanine 40 mg/m^2 days 1,2+ Novantrone 20 mg/m^2 day 1<br>3. NOVE: Mitoxantrone 10 mg/m2/d days 1-5 + etoposide 100 mg/m2/d days 1-5<br>4. Cytarabine 100 mg/m2/d days 1-5<br>5. Unknown

Supplementary Table 8. Best response according to the induction schedule in the matched cohorts.

**Induction** N(%)	**CR/CRi**	**PR**	**Resistance**	**Death within 60 days of CT**
**Historical control**	53/99 (54)	7/99 (7)	24/99 (24)	15/99 (15)
**CPX-351**	31/52 (59)	4/52 (8)	6/52 (12)	11/52 (21)

CR: complete remission. CRi: complete remission with incomplete peripheral blood recovery. PR: partial remission.

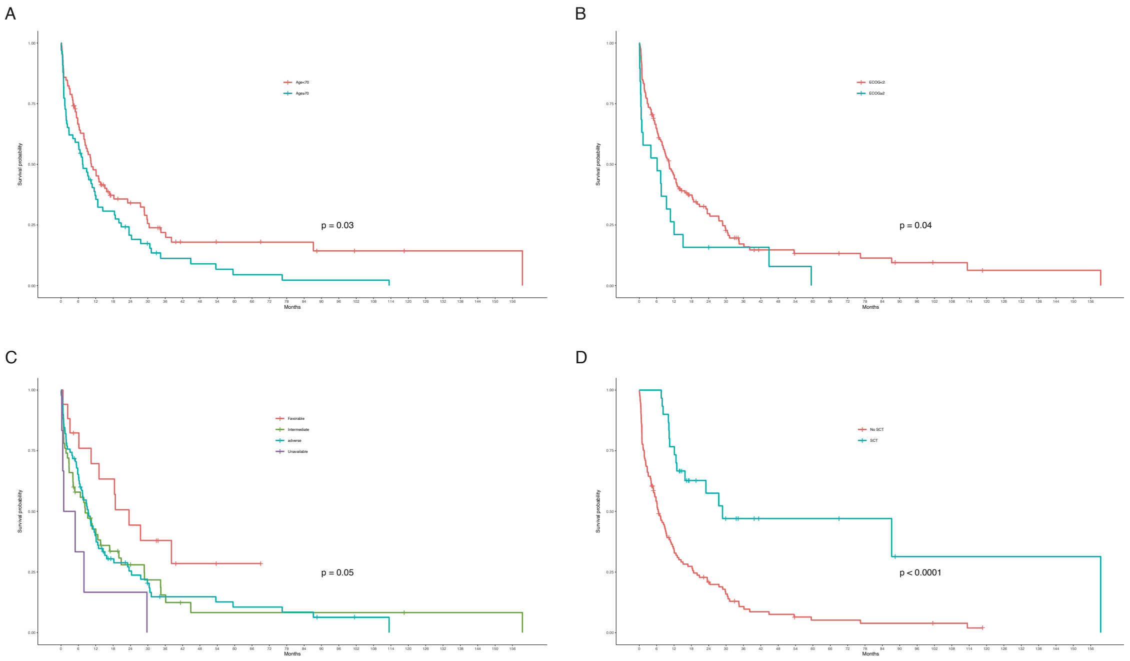

Supplementary Figure 3. Univariable analysis of overall survival (OS) in the matched cohorts. A) OS according to age; B) OS according to ECOG-Performance Status; C) OS according to European Leukemia Net 2017 (ELN17) genetic risk; D) OS according to Stem cell Transplant.

Supplementary Table 9. Mean Standard Differences (MSD) of covariates after matching considering only patients treated with standard 3/7 scheme.

**Baseline characteristics**	**After**
**Age**	-0.09
**Sex**	-0.05
**ECOG ≥2**	0.06
**HCTCI ≥3**	-0.07
**ELN17 (%)**	
• Favorable	-0.04
• Intermediate	0.03
• Adverse	0.03
• NA	0.02

ECOG: Eastern Cooperative Oncology Group. ELN17: European Leukemia Net 2017 genetic risk classification.

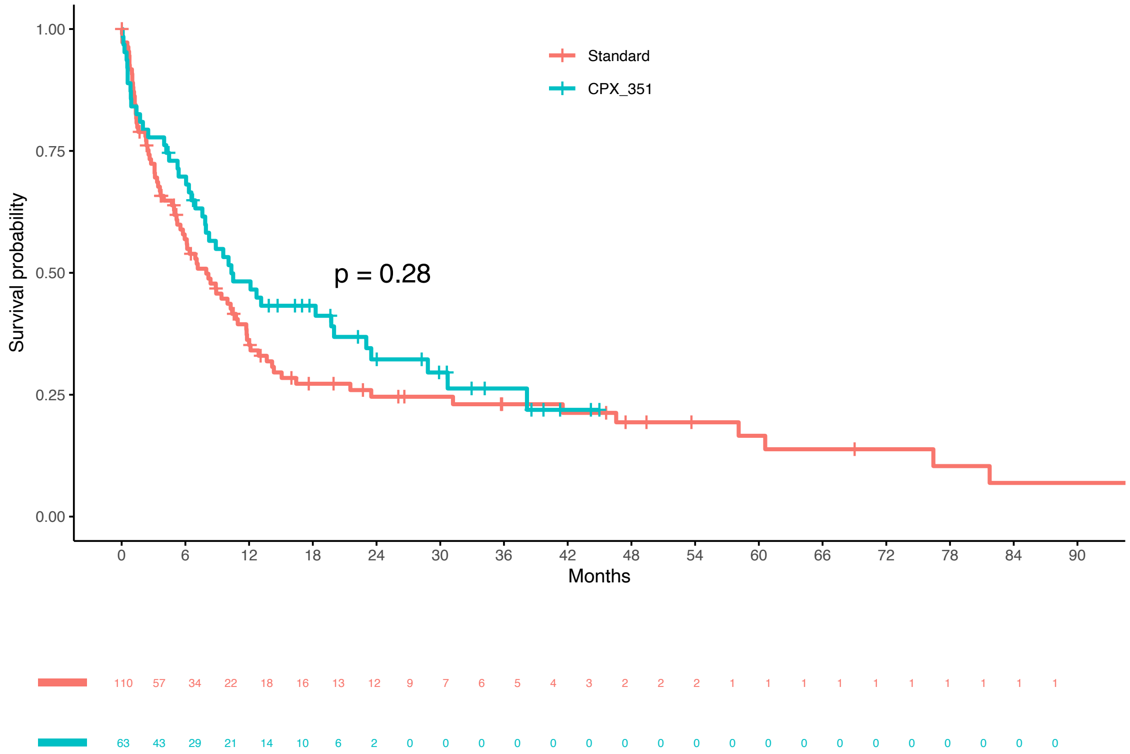

Supplementary Figure 4. Univariable analysis of overall survival (OS) in the matched cohorts considering only patients treated with standard 3+7 scheme.

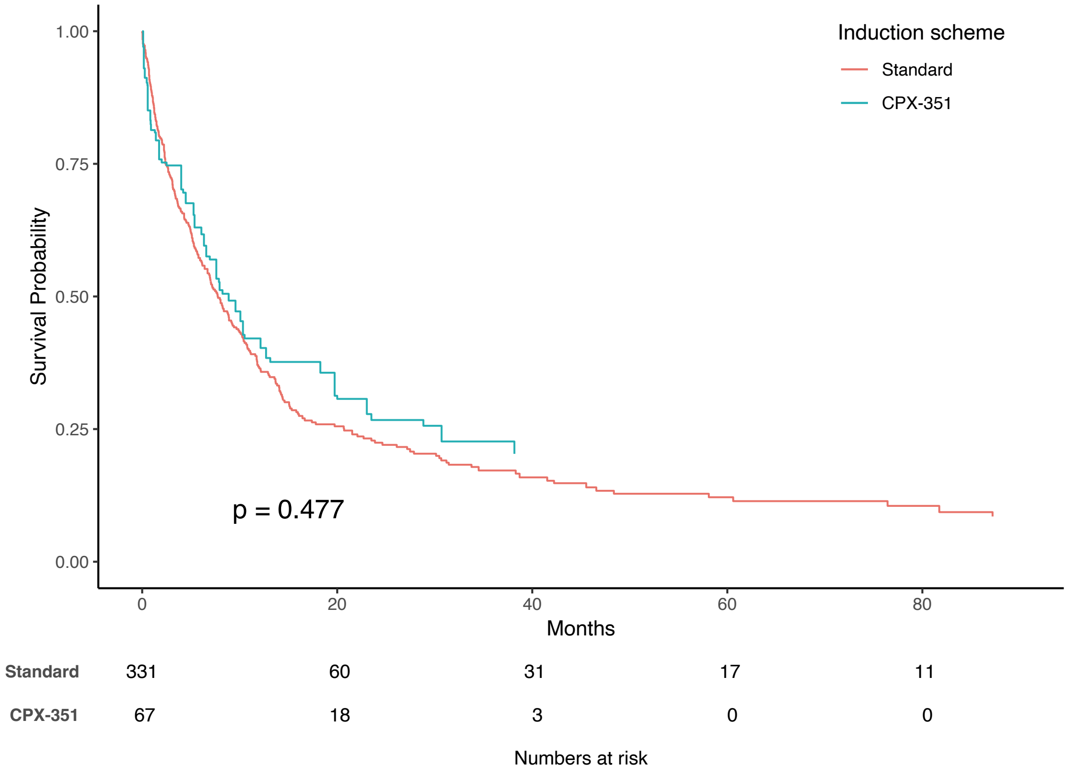

Supplementary Figure 5. OS between CPX-351 and 3+7 cohorts after inverse probability of treatment weighting analysis.
